# Supplementary material for: Enhanced Nitric Oxide Synthesis Through Nitrate Supply Improves Drought Tolerance of Sugarcane Plants
Source: Front Plant Sci. 2020 Jun 30;11:970. doi: 10.3389/fpls.2020.00970 (PMC7339982; doi:10.3389/fpls.2020.00970)
Supplement: Supplementary file 1 [file DataSheet_1.pdf]

Supplementary Material

1 Supplementary Figures

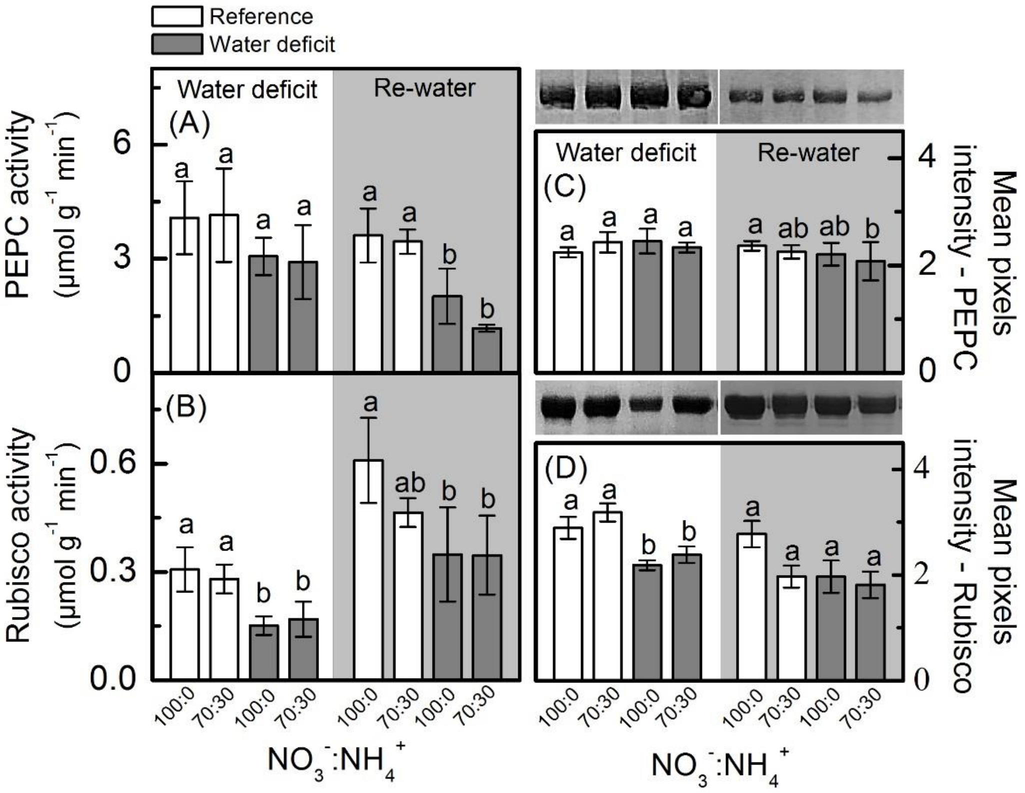

Supplementary Figure 1. *In vitro* activity of phosphoenolpyruvate carboxylase (PEPC) (A) and ribulose-1,5-bisphosphate carboxylase/oxygenase (Rubisco) (B), and immunoblots of total leaf proteins probed with antibodies against PEPC (C) and Rubisco (D) and their relative abundances (given by pixel intensity) in sugarcane plants maintained well-hydrated (reference, white bars) or subjected to water deficit (gray bars) and supplied with varying NO_3^-:NH_4^+ ratios: 100:0 and 70:30. The white area indicates the period of water deficit and the shaded area indicates the period of re-water. Bars represent the mean value of four replications ± se. Different letters indicate statistical difference among treatments (Tukey test, $p<0.05$).

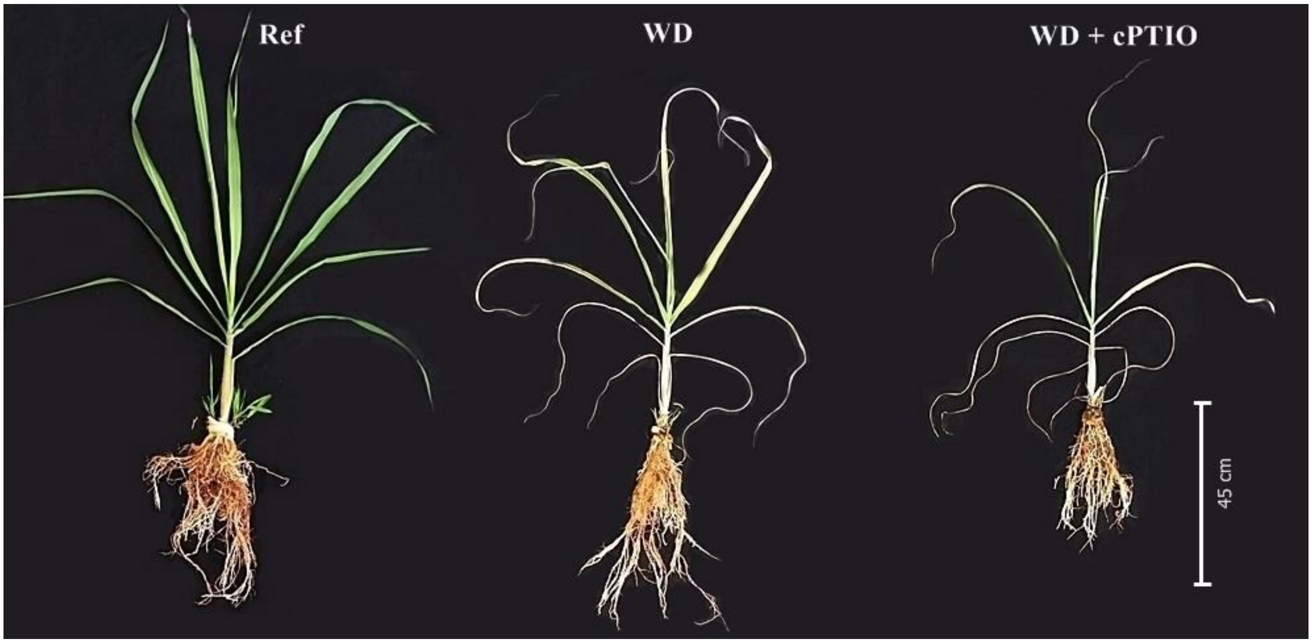

Supplementary Figure 2. Visual aspect of sugarcane plants at the end of the experiment II. Ref: plants maintained well-hydrated; WD: plants subjected to water deficit; WD+cPTIO: plants subjected to water deficit and sprayed with cPTIO. All treatments were supplied with 100:0 NO_3^-:NH_4^+.

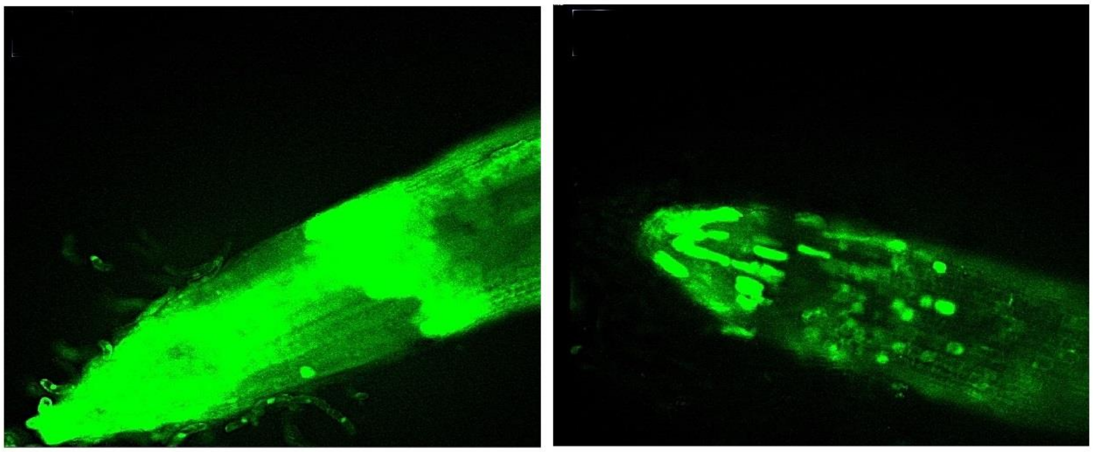

Supplementary Figure 3. Confocal microscopy images showing intracellular NO synthesis in apical sections of sugarcane roots under water deficit and supplied with 100:0 NO_3^-:NH_4^+.
